# Gender Differences in Medication Use: A Drug Utilization Study Based on Real World Data

**DOI:** 10.3390/ijerph17113926

**Published:** 2020-06-01

**Authors:** Valentina Orlando, Sara Mucherino, Ilaria Guarino, Francesca Guerriero, Ugo Trama, Enrica Menditto

**Affiliations:** 1 CIRFF, Center of Pharmacoeconomics and Drug utilization Research, University of Naples Federico II, 80131 Naples, Italy; ilaria.guarino@cirff.it (I.G.); francesca.guerriero@cirff.it (F.G.); enrica.menditto@unina.it (E.M.); 2 Department of Pharmacy, University of Naples Federico II, 80131 Naples, Italy; 3 Regional Pharmaceutical Unit, Campania Region, 80131 Naples, Italy; ugo.trama@regione.campania.it

**Keywords:** gender difference, real-world data, drug utilization study, prescription pattern, medication use, drug cost

## Abstract

A gender-specific drug utilization study was performed in the Campania region, Southern Italy. Data were based on outpatient drug prescriptions collected from administrative databases. The study population included all patients with at least one drug prescription in 2018. Prevalence was used as a measure to estimate the degree of exposure to drugs. A total of 3,899,360 patients were treated with at least one drug (54.2% females). The number of prescriptions was higher in females than males (55.6% vs. 44.4%). Females recorded higher prevalence for the majority of therapeutic groups (ATC II—anatomical therapeutic chemical), as well as for anti-inflammatory and antirheumatic products drugs (M01) (25.6% vs. 18.7%, risk ratio (RR): 0.73), beta blocking agents (C07) (14.5% vs. 11.6%, RR: 0.80), psychoanaleptics (N06) (7.1% vs. 3.7%, RR: 0.52), and antianemic preparations (B03) (2.8% vs. 6.7%, RR: 0.4). Higher prevalence was identified for males only for drugs used in diabetes (A10) (6.8% vs. 6.2%, RR: 1.1), particularly for biguanides (A10BA). Conversely, treatment duration was longer among males, explaining the higher mean cost per treated patient. This real-world study showed substantial gender differences in terms of medication use and duration of treatment and costs. These results are relevant to promoting and supporting the emerging role of precision and personalized medicine.

## 1. Introduction

Drugs are an important and indispensable tool for the prevention, diagnosis, treatment, and rehabilitation of patients. For a rational use of the drug, it is necessary that patients receive medications appropriate to their clinical needs, in doses that meet their own individual requirements, for an adequate period of time, and at the lowest cost to them and the community [1]. Therefore, the ultimate goal of a rational medical prescription is to optimize the therapeutic results, while also keeping in mind the individual characteristics of the patients, such as sex and gender differences [2,3,4,5,6].

According to the World Health Organization, sex refers to the biological and physiological characteristics that define men and women. Gender refers to the socially constructed roles, behaviors, activities, and attributes that a given society considers appropriate for men and women [7]. While it is evident that biological differences, commonly referred to as ‘sex differences’, should be considered when prescribing medicines, it is unclear to what extent sociocultural differences, commonly referred to as ‘gender differences’, should be considered by the prescribing physician [8].

Indeed, while sex differences in drug use have been demonstrated in some therapeutic areas, there is a lack of overviews regarding gender differences concerning the use of drugs in an entire population [9,10,11].

Several studies have shown that females generally use medicines more often than males [12,13,14] and the results of an interview survey conducted in 2009 showed that the rate of contact with health professionals in the previous 6 months was higher among females than males [15]. Moreover, some studies which analyzed the influence of age and gender on drug prescription in primary health care have shown that these factors condition the prescription patterns and, consequently, also their cost [16,17,18]. Particularly, prescriptions adjusted for age and gender explain approximately 35% of cost variations between medical practices, and up to 66% in the case of analysis of the therapeutic groups [12,18]. Therefore, these findings suggest the need to assess the association of gender in the context of primary care services. Providing information based on real-world data may be a useful way to explore the existing gender differences in therapeutic dynamics in terms of drug utilization and drug cost within a specific context and to optimize the use of health care resources. For this purpose, pharmacy records are a valuable resource for exploring the epidemiology of drug prescription in a population-based sample [19].

Dispensed prescriptions are a measure of drug exposure and can describe the marketing, distribution, prescription, and use of drugs in a society, with special emphasis on the medical, social, and economic consequences [20]. The aim of this study was to describe gender differences with respect to the prevalence of drug use and cost in the real-world context of Campania region, Italy.

## 2. Methods

A descriptive cross-sectional drug utilization study was performed using administrative health-related databases in the primary care setting of the Campania region, one of the largest Italian regions situated in the South of the country and representing approximately 10% of the Italian population (i.e., 5.9 million inhabitants). All the details relating to data sources have been published in previous drug utilization studies [21,22,23,24,25,26]. Particularly, the data used for this study were obtained from: (i) civil registry containing demographic information (i.e., age, gender, date of death or emigration) of all residents covered by the regional health system; and (ii) pharmaceuticals database containing information, such as the patient identification code, drug code, dose, formulation, number of packages, date of prescription, date of dispensation, and drug price.

Data sources listed were matched by record-linkage analysis through a unique and anonymous personal identification code. In Italy, such studies do not require ethical approval by an Ethics Committee as per the Italian Health Ministry/Italian Drug Agency decree of the 3rd August 2007. Furthermore, the anonymous data file is routinely used by the regional health authority for epidemiological and administrative purposes.

The study population included all patients with at least one drug prescription during the study period (1 January 2018 to 31 December 2018). Drugs included in the analysis were classified according to the anatomical therapeutic chemical (ATC) classification system [27].

All pharmacological groups analyzed were selected using the following procedure:

1. All 84 ATC second-level groups available on the Campania market were identified.
2. Twenty ATC second-level groups with a prevalence rate >3% were selected for the study.
3. From these 20 ATC second-level groups, all 57 pharmacological/therapeutic subgroups (ATC IV) accounting for >90% of the total volume expressed in defined daily dose (DDD) were selected.

### 2.1. Drug Utilization Indicators

Drug consumption was expressed as DDD. DDD is the assumed average maintenance dose per day for a drug used as per its main indication in adults and provides a fixed unit of measurement independent of dosage form (e.g., tablet strength). The value of DDD was reported as DDD/treated patient to estimate the average number of days of therapy, and it was calculated as the ratio between the total DDD consumed and the total number of patients who received at least one prescription during the study period [28]. Patients receiving at least one prescription of a drug were defined as prevalent users. Prevalence of use was evaluated per calendar year and calculated as the number of prevalent users divided by the number of all resident patients alive in the same year. Prevalence rates were expressed as percentage and stratified by year, age group, and sex. The number of sporadic users, defined as patients who received a single prescription throughout the study period, was estimated for each ATC group (ATC II). The annual cost of drug use was calculated by multiplying boxes prescribed during the year by the unit cost at the time of prescription. Drug cost was expressed in Euros as cost per treated patient. As shown in the Supplementary materials, an analysis of the chemical subgroups (ATC IV) that had a relative risk ratio (RR) of ± 20% was performed.

### 2.2. Statistical Analysis

Differences in the prevalence rate between males and females were expressed as crude and age-adjusted RR with 95% confidence interval (CI) (ratio of the prevalence in females and males). Age standardization was performed by direct standardization, where the Italian population recorded on 1 January 2018 (29,427,607 males, 31,056,366 females, 60,483,973 total, according to http://demo.istat.it/pop2018/index.html [29]) was used as the standard population. Age was categorized into the following groups: 0–6, 7–14, 15–24, 25–34, 35–44, 45–54, 55–64, 65–74, 75–84, and ≥ 85 years.

Ninety-five percent CI of crude and age-adjusted Risk Ratios (RRs) were computed using standard methods [30]. Data management was performed with Microsoft SQL server (version 2018) (Penton, USA, Fort Collins, Colorado), and all analyses were performed using the SPSS software version 17.1 for Windows (SPSS Inc., Chicago, IL, USA). A *p*-value of <0.05 denoted statistically significant differences.

## 3. Results

A total of 3,899,360 patients received at least one prescription of drugs, of whom 54.2% were females and 45.8% were males and the number of prescriptions was greater in females than males (55.6% vs. 44.4%, respectively). However, regarding the average number of days on therapy, males were treated for longer time than females (495.6 vs. 481.5 days). The prevalence of drug prescription was 66.9% overall (70.8% for females and 62.8% for males). Baseline characteristics of the study sample are shown in Table 1.

**Figure 1:**
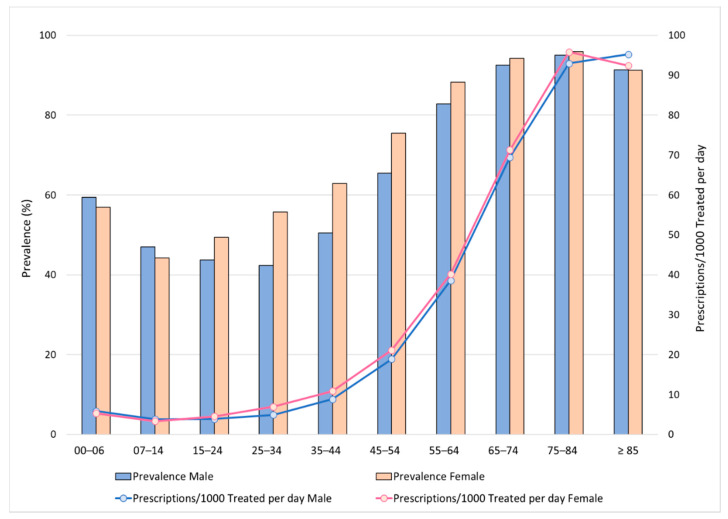
Prevalence (%) and prescriptions per 1000 treated patients per day.

As shown in Figure 1, the prevalence and prescription rates per 1000 treated patients per day for both males and females progressively increased with age. Specifically, in all age groups, the prevalence was higher in females than males, except in the age groups 0–14 years and >85 years, in which males had a higher prevalence rate than females. Particularly, among children aged 0–14 years, the prevalence rate was higher in males than females (59.4% vs. 56.0%, respectively, in the age group 0–6 years; 47% vs. 44.2%, in the age group 7–14 years). The same trends were also recorded for prescription per 1,000 treated patients per day (5.86 in males and 5.33 in females). While, among adults aged 15–64 years, the prevalence rate was higher in females than males, particularly in the age group 25–34 years (55.8% vs. 42.3%) and 35–44 years (62.9% vs. 50.5%). The same occurred for the number of prescriptions per 1,000 treated patients per day higher in females. Among older adults, aged 65–74 and 75–84 years, the prevalence rate was also higher in females than males (94.2% vs. 92.6% and 95.9% vs. 95.1%,), as well as the number of prescriptions per 1000 treated patients per day (71.3% vs. 69.5%, and 95.8% vs. 92.9%, in the age group 75–84 years). The prevalence rate was inverted in patients aged ≥85 years (91.4% for males and 91.3% for females).

Table 2 shows gender difference in prevalence rates (over than 3%) of therapeutic groups (ATC II—anatomical therapeutic chemical), which, in the majority of cases, recorded higher values for females than males.

Particularly, the prevalence rate of anti-inflammatory and antirheumatic products drugs (M01) was higher in females than males (25.6% vs. 18.7%, RR: 0.73). Moreover, focusing on the pertaining chemical subgroup (ATC IV), coxib drugs (M01AH) had the higher gender difference in prevalence, higher in females (4.2% vs. 2.1%, RR: 0.51) (Supplementary Table S1). The same trend, with higher prevalence recorded for females, was observed for the majority therapeutic groups, such as for beta blocking agents (C07) (14.5% vs. 11.6%, RR: 0.80); psychoanaleptics (N06) (7.1% vs. 3.7%, RR: 0.52), more specifically for the pertaining other antidepressants (N06AX) and selective serotonin reuptake inhibitors (N06AB); antianemic preparations (B03) (2.8% vs. 6.7%, RR: 0.4), particularly for folic acid and derivatives (B03BB) and iron bivalent, oral preparations (B03AA); and analgesics (N02) (2.8% vs. 5.5%, RR: 0.51), particularly for selective serotonin agonists (N02CC).

Gender differences in terms of prevalence rates markedly higher for females were recorded for vitamins (A11) (4.4% vs. 20.5%, RR: 0.21), specifically for vitamin D and analogues (A11CC) and thyroid therapy (H03) (1.5% vs. 6.5%, RR: 0.23), specifically for thyroid hormones (H03AA).

An opposite trend, recording higher prevalence rate for males than females, was identified for drugs used in diabetes (A10) (6.8% vs. 6.2%, RR: 1.1), particularly for biguanides (A10BA). Supplementary Table S1 details gender differences in prevalence and risk ratio related to the pertaining chemical subgroup (ATC IV).

Table 3 shows the treatment intensity and cost per treated patient for each therapeutic group (ATC II) included in this study. The same analysis was performed for the pertaining chemical subgroup (ATC IV) and is shown in Supplementary Table S2.

Particularly, the proportion of sporadic users of anti-inflammatory and antirheumatic products (M01) was higher for males than females (54.4% vs. 47.1%), as observed for the corresponding coxibs (ATC IV: M01AH) (73.0% vs. 65.5%). Indeed, differences in terms of cost for patients were observed for this therapeutic group, recording higher cost for females than males (EUR 12.3 vs. EUR 10.1), as well as for DDD per treatment (44.6 vs. 37.5). Similar trend was observed for corticosteroids for systemic use (H02), beta blocking agents (C07) and psychoanaleptics (N06).

The case of analgesics (N02) was different, recording a higher proportion of sporadic users and higher cost for males than females (61.1% vs. 53.9% and EUR 74.5 vs. EUR 71.3) but DDD per treatment was lower than females (21.5 vs. 23.2), as was the case for the majority of pertaining chemical subgroups (ATC IV) such as selective serotonin agonists (N02CC), other opioids (N02AX), salicylic acid and derivatives (N02BA).

Conversely, for lipid modifying agents (C10), higher proportions of sporadic users were identified for females (10.6% vs. 11.2%), subsequently recording a lower DDD per treatment (221.2 vs. 273.6) and lower costs (EUR 92.5 vs. EUR 114.3) than males. This was markedly shown for the pertaining chemical subgroup (ATC IV) and other lipid modifying agents (C10AX). A similar trend was observed for antianemic preparations (B03) and antiepileptics (N03).

## 4. Discussion

This population-based drug utilization study reports information on gender differences in medication use providing up-to-date data in an Italian setting.

The main finding arising from our analysis was that females showed a higher prevalence of drug use and prescriptions, while males had longer duration of treatment, resulting in higher costs than females. These findings are in line with other studies, as reported from results of a Catalan study, recording higher prevalence of drug use in women (51.1%), suggesting that they were probably likely to make more visits to their doctor. [12] Corroborating with our results, another population-based study conducted in US reported that women were significantly more likely than men to use one or more medications (68% vs. 59%) [31]. These results were also confirmed by previous Italian and German studies [32,33].

The larger number of prescriptions observed for females is often due to the higher rates of contact with the health service [12] and higher life expectancy at birth than males (85.2 vs. 80.8 years, respectively). According to the Italian Medicine Agency, the prevalence rate increases with aging from approximately 50% in children to >95% in the elderly [32]. Furthermore, for females the highest prevalence among most therapeutic groups (ATC II) was also recorded, while males showed a higher prevalence only for antidiabetic drugs. Indeed, the treatment duration related to antidiabetic drugs was consistent with the chronic use; in fact, patients were treated for at least 9 months per year and 8.4% of the male population received only one prescription during the year. These results are in line with the evidence existing in literature, as the higher proportion observed in males may be explained by the higher prevalence of diabetic disease [33]. Furthermore, use of drugs for acid-related disorders (A02) and specifically proton pump inhibitors (A02BC) was more common among females, and this is in agreement with a study conducted in Netherlands reporting 57.1% of females Proton Pump Inhibitors (PPIs) users [34]. These results are probably due to a greater use of anti-inflammatory and antirheumatic drugs (M01) in females than males. A study conducted by Davis et al. [35] revealed that females were more likely to regularly use nonsteroidal anti-inflammatory drugs than males.

Indeed, use of anti-inflammatory and antirheumatic drugs was prevalent in females compared with males (+7%), and these results are in agreement with the higher prevalence of musculoskeletal system and rheumatic disorders observed in females [32,36]. In addition, these drugs were used for short periods, and each female user was treated for approximately 45 days on average, while 47% received only one prescription.

Approximately, 21% of females used vitamins (A11), especially vitamin D, and this value was higher than that recorded for males (+16%). This result was not surprising, considering that the use of antiosteoporosis drugs mainly concerns females worldwide [6,37,38]. Another relevant result of our study concerned drugs that affect the nervous system (antidepressants and analgesics). Females users of psychoanaleptic drugs (N06), such as antidepressant (N06AX) and selective or nonselective serotonin reuptake inhibitors (N06AB–N06AA), more than males; it is likely that this observation is related to the hormonal profile and gender differences in serotonin activities [38]. Indeed, several studies at primary care have reported higher proportions of depressed females [39].

Regarding analgesic drugs (N02), it was observed that more prescriptions were dispensed for females than males. Also, females used more over-the-counter analgesics than males [40], as demonstrated by evidence from a large telephone interview study. In that study, a significantly higher proportion of females than males self-medicated for pain [41].

In our study, another relevant gender difference was recorded for thyroid preparations (H03). Females presented greater use of these drugs (+4.95%); this finding is consistent with the higher prevalence of thyroid disease in females (2–7-fold higher) than males [42].

In agreement with previous findings regarding cardiovascular drugs, in the present study, angiotensin-converting enzyme (ACE) inhibitors (C09AA) and another lipid-modifying agent (C10AX) showed a higher tendency of being prescribed in males than females [43].

On the other hand, females are less likely to receive preventive care treatments (i.e., lipid-modifying agents and ACE inhibitors) and be treated less aggressively. In fact, ACE inhibitors and diuretics drugs (C09BA), angiotensin II receptor blockers and diuretics drugs (C09DA), sulfonamides drugs (C03CA), beta-blocking agents selective and thiazides drugs (C07BB) showed a higher tendency of being prescribed in females than males. The higher prevalence rate of diuretics drugs in females may be due to the higher prevalence of heart failure observed in females [33].

More detailed patient information, mainly regarding clinical outcomes such as the presence of one or more chronic conditions, were not available at the time of analyses. The lack of clinical information on patients in order to assess the reason behind the observed differences represents the principal limitation of the present study. Despite this, we were able to delineate a population-based overview of drug use giving a solid background for further analyses to be implemented with clinical outcomes. It is important to emphasize that gender differences may only be hypothesized from these data. Moreover, data on out-of-pocket drugs were excluded.

Nevertheless, our findings support those of previous international and Italian studies [44,45,46,47,48,49,50]. Thus, data obtained from a real-world context are a powerful starting point of view for analyses of gender differences in treatment patterns. The pharmacy administrative database includes demographic elements; however, sociodemographic variables (e.g., education, economic status) and clinical information (e.g., diagnosis), are not included, representing a limitation of this approach.

This initiative provided preliminary data; further analysis concerning specific age groups is warranted to understand the different patterns of drug use.

## 5. Conclusions

In this cross-sectional study on gender differences in medication use, females tended to receive more drugs than males, and appeared to be less sporadic users compared with males. At the same time, costs related to the treatment of females, which are incurred by the Italian National Health Service, were lower. Our goal in this study was to raise awareness regarding gender differences in the use of prescription medication. These real-world data, highlighting gender differences based on the prescribing habits of doctors, may be relevant to promote and support the emerging role of precision and personalized medicine. In this context, real-world data represents an essential tool evaluating and directing novel therapies towards personalized medicine, as only through scientific evidence regarding gender and sex differences it is possible to guarantee the best care service and adequacy of health interventions.

## Figures and Tables

**Table 1:** Characteristics of the study population.

Baseline Characteristics	Male	Female	Overall
Number of treated patients	1,785,518 (45.8%)	2,113,842 (54.2%)	3,899,360
Prevalence of drug use/100 inhabitants (95% CI)	62.84 (62.75–62.94)	70.79 (70.70–71.89)	66.92 (66.85–67.00)
Number of prescriptions	25,454,363 (44.4%)	31,903,087 (55.6%)	57,357,450
DDD per treated patient	495.6	481.5	488.0
Cost per treated patient (€)	227.2	225.6	226.3

Abbreviations: CI, confidence interval; DDD, defined daily dose.

**Table 2:** Gender differences in prevalence (%) of drug therapy (therapeutic groups—ATC II).

ATC II	Description	Adjusted Prevalence % (CI)			Adjusted RR (95% CI) Male/Female
		Male	Female	Overall	
J01	Antibacterials for systemic use	43.33 (43.26−43.41)	50.55 (50.48−50.64)	47.50 (47.45−47.56)	0.857 (0.856−0.858)
A02	Drugs for acid related disorders	24.96 (24.90−25.02)	30.88 (30.81−30.94)	28.45 (28.41−28.50)	0.808 (0.807−0.810)
M01	Anti-inflammatory and antirheumatic products	18.67 (18.61−18.71)	25.66 (25.60−25.72)	22.55 (22.51−22.59)	0.727 (0.726−0.728)
C09	Agents acting on the renin–angiotensin system	22.16 (22.10−22.22)	23.48 (23.42−23.54)	23.25 (23.21−23.29)	0.943 (0.942−0.946)
R03	Drugs for obstructive airway diseases	15.37 (15.31−15.42)	17.02 (16.98−17.07)	16,40 (16.36−16.43)	0.902 (0.901−0.905)
H02	Corticosteroids for systemic use	13.59 (13.55−13.64)	16.81 (16.77−16.86)	15.41 (15.38−15.44)	0.808 (0.807−0.810)
C10	Lipid modifying agents	14.00 (13.95−14.05)	14.31 (14.27−14.36)	14.41 (14.38−14.45)	0.977 (0.976−0.980)
C07	Beta blocking agents	11.57 (11.53−11.61)	14.53 (14.49−14.58)	13.34 (13.31−13.37)	0.796 (0.794−0.798)
A11	Vitamins	4.43 (4.41−4.46)	20.54 (20.49−20.59)	12.94 (12.92−12.98)	0.215 (0.215−0.216)
B01	Antithrombotic agents	10.73 (10.69−10.77)	12.00 (11.96−12.04)	11.65 (11.62−11.68)	0.894 (0.892−0.896)
R06	Antihistamines for systemic use	7.55 (7.52−7.59)	9.73 (9.70−9.77)	8.74 (8.72−8.77)	0.776 (0.774−0.779)
A07	Antidiarrheals. Intestinal anti-inflammatory/anti-infective agents	6.44 (6.41−6.47)	7.37 (7.34−7.40)	7.02 (6.99−7.04)	0.873 (0.871−0.877)
A10	Drugs used in diabetes	6.80 (6.77−6.83)	6.25 (6.22−6.28)	6.65 (6.62−6.67)	1.088 (1.085−1.092)
C08	Calcium channel blockers	6.38 (6.35−6.42)	6.48 (6.45−6.51)	6.57 (6.55−6.59)	0.985 (0.982−0.989)
N06	Psychoanaleptics	3.66 (3.64−3.68)	7.07 (7.04−7.10)	5.51 (5.49−5.53)	0.517 (0.515−0.519)
C03	Diuretics	4.27 (4.24−4.29)	6.31 (6.28−6.34)	5.47 (5.45−5.49)	0.676 (0.673−0.679)
B03	Antianemic preparations	2.77 (2.75−2.79)	6.72 (6.69−6.75)	4.89 (4.87−4.91)	0.412 (0.410−0.414)
H03	Thyroid therapy	1.52 (1.51−1.54)	6.47 (6.44−6.50)	4.13 (4.11−4.14)	0.235 (0.234−0.236)
N02	Analgesics	2.80 (2.77−2.81)	5.50 (5.46−5.52)	4.25 (4.24−4.28)	0.509 (0.507−0.511)
N03	Antiepileptics	2.97 (2.95−2.99)	3.80 (3.77−3.82)	3.44 (3.43−3.46)	0.781 (0.777−0.785)

Abbreviations: ATC, Anatomical Therapeutic Chemical Classification System II level, CI, confidence interval; RR, risk ratio.

**Table 3:** Gender difference in DDD/treated, sporadic users (%) and cost for treated of drug therapy (ATC II).

ATC II	Description	DDD/Treated			Sporadic Users (%)			Cost/Treated (€)		
		Male	Female	Overall	Male	Female	Overall	Male	Female	Overall
J01	Antibacterials for systemic use	18.6	18.8	18.7	47.6	43.9	45.6	29.1	28.8	28.9
A02	Drugs for acid related disorders	110.1	110.1	110.1	26.5	26.1	26.3	64.1	63.1	63.5
M01	Anti-inflammatory and antirheumatic products	37.5	44.6	41.7	54.4	47.1	50.1	10.1	12.3	11.4
C09	Agents acting on the renin–angiotensin system	368.2	350.8	359.1	6.8	6.8	6.8	80.3	79.4	79.9
R03	Drugs for obstructive airway diseases	83.8	70.9	76.9	51.8	54.9	53.5	118.8	95.0	106
H02	Corticosteroids for systemic use	29.0	30.8	30	68.4	65.4	66.7	7.8	8.6	8.2
C10	Lipid modifying agents	273.6	221.2	246.6	10.6	11.2	10.9	114.3	92.5	103.0
C07	Beta blocking agents	155.0	168.4	162.6	11.0	10.6	10.8	38.6	39.4	39.0
A11	Vitamins	86.2	97.8	95.8	37.6	24.2	26.5	40.4	51.1	49.3
B01	Antithrombotic agents	189.9	174.2	181.5	21.7	24.0	22.9	38.6	44.7	41.9
R06	Antihistamines for systemic use	57.3	59.3	58.4	63.9	63.1	63.4	13.4	13.5	13.5
A07	Antidiarrheals. Intestinal anti-inflammatory	41.5	34.1	37.5	60.6	58.1	59.2	58.1	50.7	54.1
A10	drugs used in diabetes	271.2	267.4	269.3	8.4	8.5	8.5	123.2	124.3	123.7
C08	Calcium channel blockers	297.3	267.8	282.2	14.4	16.9	15.7	61.5	59.6	60.5
N06	Psychoanaleptics	182.4	192.2	189	26.5	22.7	24.0	81.8	82.0	81.9
C03	Diuretics	175.7	149.2	159.7	32.1	34.3	33.4	25.8	22.2	23.6
B03	Antianemic preparations	116.7	100.7	105	48.9	53.2	52.1	24.6	20.2	21.4
H03	Thyroid therapy	160.3	147.0	149.4	24.0	19.8	20.6	17.9	17.7	17.7
N02	Analgesics	21.5	23.2	22.6	61.1	53.9	56.2	74.5	71.3	72.3
N03	Antiepileptics	142.8	111.3	124.9	24.7	27.0	26.0	179.0	151.4	163.3

## Supplementary Materials

The following are available online at https://www.mdpi.com/1660-4601/17/11/3926/s1, Table S1: Gender difference in prevalence (%) and Risk Ratio of drug therapy (ATC IV), Table S2: Gender difference in DDD/Treated and Costs/Treated of drug therapy (ATC IV).

## References

[1] World Health Organization The rational use of drugs. Proceedings of the WHO Report of the Conferences of Experts.

[2] Soldin O.P., Chung S.H., Mattison D.R. (2011). Sex differences in drug disposition. J. Biomed. Biotechnol..

[3] Regitz-Zagrosek V. (2012). Sex and gender differences in health. Science & Society Series on Sex and Science. EMBO Rep..

[4] Gulbins H., Vogel B., Reichenspurner H. (2013). Gender effects on health care costs in cardiovascular medicine-a black box?. Thorac. Cardiovasc. Surg..

[5] Owens G.M. (2008). Gender differences in health care expenditures, resource utilization, and quality of care. J. Manag. Care Pharm..

[6] Putignano D., Bruzzese D., Orlando V., Fiorentino D., Tettamanti A., Menditto E. (2017). Differences in drug use between men and women: An Italian cross sectional study. BMC Women’s Health.

[7] World Health Organization What Do We Mean by “Sex” and “Gender”. http://www.who.int/gender/whatisgender/en.

[8] Loikas D., Wettermark B., von Euler M., Bergman U., Schenck-Gustafsson K. (2013). Differences in drug utilisation between men and women: A cross-sectional analysis of all dispensed drugs in Sweden. BMJ Open.

[9] Campbell C.I., Weisner C., Leresche L., Ray G.T., Saunders K., Sullivan M.D., BantaGreen C.J., Merrill J.O., Silverberg M.J., Boudreau D. (2010). Age and gender trends in long-term opioid analgesic use for noncancer pain. Am. J. Public Health.

[10] Johnell K., Fastbom J. (2011). Gender and use of hypnotics or sedatives in old age: A nationwide register-based study. Int. J. Clin. Pharm..

[11] Kautzky-Willer A., Harreiter J. (2017). Sex and gender differences in therapy of type 2 diabetes. Diabetes Res. Clin. Pract..

[12] Fernández-Liz E., Modamio P., Catalán A., Lastra C.F., Rodríguez T., Mariño E.L. (2008). Identifying how age and gender influence prescription drug use in a primary health care environment in Catalonia, Spain. Br. J. Clin. Pharmacol..

[13] Roe C.M., McNamara A.M., Motheral B.R. (2002). Gender- and age-related prescription drug use patterns. Ann. Pharmacother..

[14] Glaeske G., Gerdau-Heitmann C., Hofel F., Schicktanz C. (2012). “Gender-specific drug prescription in Germany” results from prescriptions analyses. Handb. Exp. Pharmacol..

[15] Sondik E.J.M., Jennifer H., Gentleman J.F. (2010). Summary Health Statistics for the U.S. Population: National Health Interview Survey, 2009.

[16] Sleator D.J.D. (1993). Towards accurate prescribing analysis in general practice: Accounting for the effects of practice demography. Br. J. Gen. Pract..

[17] Lloyd D.C.E.F., Harris C.M., Roberts D.J. (1995). Specific therapeutic age-sex related prescribing units (STAR-PUs): Weightings for analysing general practices’ prescribing in England. BMJ.

[18] Roberts S.J., Harris C.M. (1993). Age, sex and temporary resident originated prescribing units (ASTRO-PUs): New weightings for analysing prescribing of general practices in England. BMJ.

[19] Melzer D., Tavakoly B., Winder R.E., Masoli J.A., Henley W.E., Ble A., Richards S.H. (2015). Much more medicine for the oldest old: Trends in UK electronic clinical records. Age Ageing.

[20] World Health Organization (WHO) (1977). The Selection of Essential Drugs.

[21] Iolascon G., Gimigliano F., Moretti A., Riccio I., Di Gennaro M., Illario M., Monetti V.M., Orlando V., Menditto E. (2016). Rates and reasons for lack of persistence with anti-osteoporotic drugs: Analysis of the Campania region database. Clin. Cases Miner. Bone Metab..

[22] Menditto E., Cahir C., Aza-Pascual-Salcedo M., Bruzzese D., Poblador-Plou B., Malo S., Costa E., González-Rubio F., Gimeno-Miguel A., Orlando V. (2018). Adherence to chronic medication in older populations: Application of a common protocol among three European cohorts. Patient Prefer Adherence.

[23] Menditto E., Guerriero F., Orlando V., Crola C., Di Somma C., Illario M., Morisky D.E., Colao A. (2015). Self-assessment of adherence to medication: A case study in Campania region community-dwelling population. J. Aging Res..

[24] Casula M., Catapano A.L., Piccinelli R., Menditto E., Manzoli L., De Fendi L., Orlando V., Flacco M.E., Gambera M., Filippi A. (2014). Assessment and potential determinants of compliance and persistence to antiosteoporosis therapy in Italy. Am. J. Manag. Care.

[25] Guerriero F., Orlando V., Monetti V.M., Russo V., Menditto E. (2017). Biological therapy utilization, switching, and cost among patients with psoriasis: Retrospective analysis of administrative databases in Southern Italy. Clin. Outcomes Res..

[26] Moreno-Juste A., Menditto E., Orlando V., Monetti V.M., Gimeno-Miguel A., González-Rubio F., Aza-Pascual-Salcedo M.M., Cahir C., Prados-Torres A., Riccard G. (2019). Treatment patterns of diabetes in Italy: A population-based study. Front. Pharmacol..

[27] WHOCC-ATC/DDD Index. https://www.whocc.no/atc_ddd_index/.

[28] WHO Collaborating Centre for Drug Statistics Methodology (2019). Guidelines for ATC Classification and DDD Assignment. https://www.whocc.no/news/guidelines_for_atc_classification_and_ddd_assignment.

[29] Demo-Geodemo Mappe, Popolazione, Statistiche Demografiche dell’ISTAT. http://demo.istat.it/..

[30] Newman S.C. (2001). Biostatistical Methods in Epidemiology.

[31] Manteuffel M., Williams S., Chen W., Verbrugge R.R., Pittman D.G., Steinkellner A. (2014). Influence of patient sex and gender on medication use, adherence, and prescribing alignment with guidelines. J. Womens Health.

[32] The Medicines Utilisation Monitoring Centre (2019). National Report on Medicines Use in Italy. Year 2018.

[33] Stock S.A., Stollenwerk B., Redaelli M., Civello D., Lauterbach K.W. (2008). Sex differences in treatment patterns of six chronic diseases: An analysis from the German statutory health insurance. J. Womens Health.

[34] Van Boxel O.S., Hagenaars M.P., Smout A.J.P.M., Siersema P.D. (2009). Socio-demographic factors influence chronic proton pump inhibitor use by a large population in the Netherlands. Aliment. Pharmacol. Ther..

[35] Davis J.S., Lee H.Y., Kim J., Advani S.M., Peng H.L., Banfield E., Frazier-Wood A.C. (2017). Use of non-steroidal anti-inflammatory drugs in US adults: Changes over time and by demographic. Open Heart.

[36] Santalucia P., Franchi C., Djade C.D., Tettamanti M., Pasina L., Corrao S., Mannucci P.M. (2015). Gender difference in drug use in hospitalized elderly patients. Eur. J. Intern. Med..

[37] Iolascon G., Gimigliano F., Orlando V., Capaldo A., Di Somma C., Menditto E. (2013). Osteoporosis drugs in real-world clinical practice: An analysis of persistence. Aging Clin. Exp. Res..

[38] Cawthon P.M. (2011). Gender differences in osteoporosis and fractures. Clin. Orthop. Relat. Res..

[39] Pinto-Meza A., Usall J., Serrano-Blanco A., Suárez D., Haro J.M. (2006). Gender differences in response to antidepressant treatment prescribed in primary care. Does menopause make a difference?. J. Affect. Disord..

[40] Richardson J., Holdcroft A. (2009). Gender differences and pain medication. Womens Health.

[41] Bassols A., Bosch F., Baños J.E. (2002). How does the general population treat their pain? A survey in Catalonia, Spain. J. Pain Symptom Manag..

[42] Bauer M., Glenn T., Pilhatsch M., Pfennig A., Whybrow P.C. (2014). Gender differences in thyroid system function: Relevance to bipolar disorder and its treatment. Bipolar Disord..

[43] Ljungman C., Kahan T., Schiöler L., Hjerpe P., Hasselström J., Wettermark B., Manhem K. (2014). Gender differences in antihypertensive drug treatment: Results from the Swedish Primary Care Cardiovascular Database (SPCCD). J. Am. Soc. Hypertens..

[44] Cammarota S., Bruzzese D., Catapano A.L., Citarella A., De Luca L., Manzoli L., Masulli M., Menditto E., Mezzetti A., Riegler S. (2014). Lower incidence of macrovascular complications in patients on insulin glargine versus those on basal human insulins: A population-based cohort study in Italy. Nutr. Metab. Cardiovasc. Dis..

[45] Illario M., Vollenbroek-Hutten M.M.R., Molloy D.W., Menditto E., Iaccarino G., Eklund P. (2015). Active and healthy ageing and independent living. J. Aging Res..

[46] Illario M., Vollenbroek-Hutten M.M.R., Molloy D.W., Menditto E., Iaccarino G., Eklund P. (2016). Active and healthy ageing and independent living. J. Aging Res..

[47] Coretti S., Romano F., Orlando V., Codella P., Prete S., Di Brino E., Ruggeri M. (2015). Economic evaluation of screening programs for hepatitis C virus infection: Evidence from literature. Risk Manag. Healthc. Policy.

[48] Menditto E., Orlando V., Coretti S., Putignano D., Fiorentino D., Ruggeri M. (2015). Doctors commitment and long-term effectiveness for cost containment policies: Lesson learned from biosimilar drugs. Clin. Outcomes Res..

[49] Scala D., Menditto E., Caruso G., Monetti V.M., Orlando V., Guerriero F., Buonomo G., Caruso D., D’Avino M. (2018). Are you more concerned about or relieved by medicines? An explorative randomized study of the impact of telephone counseling by pharmacists on patients’ beliefs regarding medicines and blood pressure control. Patient Educ. Couns..

[50] Ruggeri M., Manca A., Coretti S., Codella P., Iacopino V., Romano F., Mascia D., Orlando V., Cicchetti A. (2015). Investigating the generalizability of economic evaluations conducted in Italy: A critical review. Value Health.

