# Supplementary material for: Gender Differences in Medication Use: A Drug Utilization Study Based on Real World Data
Source: Int J Environ Res Public Health. 2020 Jun 1;17(11):3926. doi: 10.3390/ijerph17113926 (PMC7312791; doi:10.3390/ijerph17113926)
Supplement: Supplementary file 1 [file ijerph-17-03926-s001.pdf]

Supplementary Materials

Table S1. Gender difference in prevalence (%) and Risk Ratio of drug therapy (ATC IV)

ATC IV	Description	Prevalence Adjusted (%)			Adjusted RR (95% CI)
		Male	Female	Overall	Male/Female
**C10AX**	**Other lipid modifying agents**	2.43	1.56	2.01	1.563 (1.553 - 1.572)
**N03AG**	**Fatty acid derivatives**	0.74	0.61	0.68	1.222 (1.210 - 1.234)
**R03BB**	**Anticholinergics**	2.24	1.85	2.09	1.182 (1.180 - 1.187)
**C09AA**	**ACE inhibitors, plain**	7.16	6.15	6.77	1.165 (1.161 - 1.169)
**A10BA**	**Biguanides**	4.29	3.80	4.11	1.127 (1.122 - 1.132)
**A10BB**	**Sulfonylureas**	1.22	1.10	1.18	1.106 (1.097 - 1.115)
**N03AA**	**Barbiturates and derivatives**	0.19	0.18	0.19	1.030 (1.010 - 1.051)
**N03AF**	**Carboxamide derivatives**	0.40	0.40	0.40	1.023 (1.010 - 1.037)
**R03AC**	**Selective beta-2-adrenoreceptor agonists**	2.98	2.94	2.98	1.014 (1.009 - 1.019)
**C07AG**	**Alpha and beta blocking agents**	1.47	1.48	1.51	0.996 (0.988 - 1.003)
**C08CA**	**Dihydropyridine derivatives**	6.00	6.01	6.13	0.999 (0.996 - 1.003)
**A10AB**	**Insulins and analogues for injection, fast-acting**	1.43	1.44	1.46	0.994 (0.987 - 1.001)
**A10BD**	**Combinations of oral blood glucose lowering drugs**	0.68	0.69	0.70	0.992 (0.982 - 1.003)
**A07EC**	**Aminosalicylic acid and similar agents**	1.00	1.02	1.02	0.974 (0.966 - 0.982)
**B01AC**	**Platelet aggregation inhibitors excl. heparin**	9.01	9.46	9.47	0.953 (0.950 - 0.956)
**N03AE**	**Benzodiazepine derivatives**	0.18	0.19	0.19	0.921 (0.903 - 0.939)
**R03AL**	**Adrenergics in combination with anticholinergics**	2.61	2.76	2.71	0.945 (0.941 - 0.950)
**C10AA**	**HMG CoA reductase inhibitors**	12.08	12.91	12.74	0.936 (0.933 - 0.938)
**C09CA**	**Angiotensin II receptor blockers (ARBs), plain**	5.85	6.46	6.28	0.906 (0.902 - 0.909)
**J01CR**	**Combinations of penicillins, incl. beta-lactamase inhibitors**	20.86	23.44	22.38	0.890 (0.888 - 0.892)
**J01DD**	**Third-generation cephalosporins**	12.87	14.64	13.95	0.879 (0.877 - 0.881)
**R03AK**	**Adrenergics in combination with corticosteroids**	4.06	4.60	4.40	0.882 (0.878 - 0.885)
**R03BA**	**Glucocorticoids**	9.70	11.16	10.54	0.869 (0.867 - 0.871)
**C03DA**	**Aldosterone antagonists**	1.16	1.38	1.31	0.843 (0.836 - 0.849)
**J01FA**	**Macrolides**	11.78	14.03	13.05	0.839 (0.838 - 0.841)
**J01MA**	**Fluoroquinolones**	12.22	14.55	13.62	0.840 (0.838 - 0.842)
**C07AB**	**Beta blocking agents, selective**	8.91	10.90	10.12	0.817 (0.815 - 0.819)
**A02BC**	**Proton pump inhibitors**	22.06	27.15	25.10	0.812 (0.811 - 0.814)
**H02AB**	**Glucocorticoids**	13.59	16.82	15.41	0.808 (0.807 - 0.810)
**C09BA**	**ACE inhibitors and diuretics**	3.40	4.28	3.93	0.794 (0.791 - 0.798)
**J01CA**	**Penicillins with extended spectrum**	3.96	4.94	4.50	0.802 (0.799 - 0.805)
**R06AE**	**Piperazine derivatives**	3.95	5.05	4.56	0.782 (0.779 - 0.785)
**R06AX**	**Other antihistamines for systemic use**	4.00	5.27	4.69	0.759 (0.756 - 0.762)
**A07AA**	**Antibiotics**	4.94	6.62	5.89	0.745 (0.743 - 0.748)
**C09DA**	**Angiotensin II receptor blockers and diuretics**	5.16	7.23	6.34	0.714 (0.712 - 0.717)
**A02BX**	**Other drugs for peptic ulcer and gastro-oesophageal reflux disease**	4.34	5.99	5.25	0.724 (0.722 - 0.727)
**M01AE**	**Propionic acid derivatives**	9.83	13.75	12.00	0.715 (0.714 - 0.717)
**M01AB**	**Acetic acid derivatives and related substances**	7.51	10.55	9.20	0.712 (0.710 - 0.714)
**N02BA**	**Salicylic acid and derivatives**	0.05	0.07	0.06	0.697 (0.673 - 0.721)
**M01AX**	**Other antiinflammatory and antirheumatic agents, non-steroids**	3.97	5.69	4.92	0.697 (0.694 - 0.700)
**C03CA**	**Sulfonamides, plain**	3.34	4.91	4.28	0.680 (0.677 - 0.683)
**N03AX**	**Other antiepileptics**	1.88	2.83	2.41	0.663 (0.659 - 0.667)

**N02AB**	**Phenylpiperidine derivatives**	0.12	0.19	0.16	0.603 (0.590 - 0.617)
**C07BB**	**Beta blocking agents, selective, and thiazides**	1.04	1.72	1.41	0.608 (0.603 - 0.612)
**N06AX**	**Other antidepressants**	1.28	2.33	1.86	0.550 (0.546 - 0.553)
**N02AA**	**Natural opium alkaloids**	0.39	0.75	0.58	0.517 (0.511 - 0.522)
**N06AB**	**Selective serotonin reuptake inhibitors**	2.50	4.97	3.83	0.504 (0.501 - 0.506)
**M01AH**	**Coxibs**	2.13	4.21	3.25	0.507 (0.505 - 0.510)
**N02AX**	**Other opioids**	0.87	1.75	1.35	0.499 (0.495 - 0.502)
**N06AA**	**Non-selective monoamine reuptake inhibitors**	0.26	0.59	0.44	0.435 (0.430 - 0.441)
**N02AE**	**Oripavine derivatives**	0.03	0.07	0.05	0.421 (0.405 - 0.437)
**H03BB**	**Sulfur-containing imidazole derivatives**	0.22	0.51	0.38	0.426 (0.420 - 0.433)
**B03BB**	**Folic acid and derivatives**	1.66	3.80	2.81	0.437 (0.435 - 0.440)
**N02CC**	**Selective serotonin (5HT1) agonists**	0.25	0.72	0.50	0.349 (0.345 - 0.353)
**B03AA**	**Iron bivalent, oral preparations**	1.13	3.56	2.43	0.319 (0.317 - 0.320)
**H03AA**	**Thyroid hormones**	1.31	5.98	3.76	0.219 (0.218 - 0.220)
**A11CC**	**Vitamin D and analogues**	4.43	20.54	12.95	0.216 (0.215 - 0.216)

Abbreviations: CI, Confidence interval; RR, risk ratio.

Table S2. Gender difference in DDD/Treated and Costs/Treated of drug therapy (ATC IV)

ATC II	ATC IV		DDD/Treated			Sporadic/Users (%)			Costs/Treated (€)		
			Male	Female	Overall	Male	Female	Overall	Male	Female	Overall
**J01**	**Antibacterials for systemic use**										
	J01CA	Penicillins with extended spectrum	17.0	17.5	17.3	79.8	78.6	79.1	5.3	5.3	5.3
	J01CR	Combinations of penicillins. incl. beta-lactamase inhibitors	16.4	17.0	16.7	69.2	67.4	68.2	14.7	14.7	14.7
	J01FA	Macrolides	12.2	12.1	12.2	75.2	73.8	74.4	12.7	12.7	12.7
	J01MA	Fluoroquinolones	10.6	9.2	9.8	63.0	65.3	64.3	19.4	17.1	18.1
	J01DD	Third-generation cephalosporins	6.8	6.9	6.8	64.1	64.6	64.4	35.7	34.1	34.8
**A02**	**Drugs for acid related disorders**										
	A02BC	Proton pump inhibitors	109.3	110.1	109.7	25.9	25.4	25.6	64.5	63.4	63.9
	A02BX	Other drugs for peptic ulcer and gastro-oesophageal reflux	34.9	32.9	33.7	56.3	55.6	55.9	20.3	19.2	19.7
**M01**	**Antiinflammatory and antirheumatic products**										
	M01AH	Coxibs	36.0	40.8	39.3	73.0	65.5	67.9	14.2	16.0	15.4
	M01AE	Propionic acid derivatives	28.2	30.2	29.4	66.4	61.7	63.6	6.8	7.5	7.2
	M01AX	Other antiinflammatory and antirheumatic agents, non-steroids	28.1	30.2	29.4	65.5	61.6	63.2	4.7	5.3	5.1
	M01AB	Acetic acid derivatives and related substances	26.8	30.4	28.9	70.1	65.0	67.1	8.0	9.0	8.6
**C09**	**Agents acting on the renin-angiotensin system**										
	C09AA	ACE inhibitors. plain	457.8	414.5	437.4	13.4	16.0	14.6	45.9	44.1	45.0
	C09CA	Angiotensin II receptor blockers (ARBs). plain	361.9	353.0	357.2	13.3	14.0	13.7	66.0	65.1	65.5
	C09DA	Angiotensin II receptor blockers and diuretics	250.0	259.4	255.6	10.7	8.9	9.7	68.1	69.3	68.8
	C09BA	ACE inhibitors and diuretics	224.6	228.7	226.9	13.4	12.0	12.6	70.3	71.8	71.1
**R03**	**Drugs for obstructive airway diseases**										
	R03BB	Anticholinergics	137.2	114.5	126.6	30.9	38.5	34.4	228.9	188.1	209.8
	R03AK	Adrenergics in combination	97.6	85.9	91.2	44.7	47.9	46.4	202.8	178.7	189.7
	R03AC	Selective beta-2-adrenoreceptor agonists	60.7	52.2	56.4	69.0	72.6	70.8	32.7	24.3	28.4
	R03AL	Adrenergics in combination	38.5	32.7	35.4	75.6	81.1	78.5	59.6	31.0	44.6
	R03BA	Glucocorticoids	25.8	24.5	25.1	70.1	72.4	71.4	27.6	25.7	26.6
**H02**	**Corticosteroids for systemic use**										
	H02AB	Glucocorticoids	29.0	30.8	30.0	68.5	65.4	66.7	7.8	8.6	8.2
**C10**	**Lipid modifying agents**										
	C10AA	HMG CoA reductase inhibitors	266.3	213.2	238.4	11.0	11.6	11.3	71.3	64.9	67.9
	C10AX	Other lipid modifying agents	126.2	121.8	124.5	15.8	20.3	17.6	168.1	149.3	160.6
**C07**	**Beta blocking agents**										
	C07BB	Beta blocking agents, selective, and thiazides	224.3	234.7	230.9	15.1	12.6	13.6	45.7	47.3	46.7
	C07AB	Beta blocking agents. selective	141.0	151.8	147.0	12.6	12.4	12.5	33.3	34.8	34.1
	C07AG	Alpha and beta blocking agents	131.3	126.3	128.8	10.3	11.6	11.0	57.6	54.5	56.0
**A11**	**Vitamins**										
	A11CC	Vitamin D and analogues	86.1	97.8	95.8	37.6	24.2	26.5	40.4	51.1	49.3
**B01**	**Antithrombotic agents**										
	B01AC	Platelet aggregation inhibitors excl. heparin	210.1	202.6	206.2	17.4	17.9	17.7	19.6	18.1	18.8
**R06**	**Antihistamines for systemic use**										
	R06AX	Other antihistamines for systemic use	62.0	63.5	62.9	64.4	64.0	64.2	15.1	15.3	15.2

	R06AE	Piperazine derivatives	46.9	48.1	47.6	69.5	69.3	69.4	10.3	10.1	10.2
**A07**	**Antidiarrheals. intestinal antiinflammatory/antiinfective agents**										
	A07EC	Aminosalicylic acid and similar agents	163.6	131.0	146.7	45.5	48.1	46.8	182.2	138.6	159.6
	A07AA	Antibiotics	15.7	16.3	16.1	64.8	61.0	62.6	31.1	32.0	31.6
**A10**	**Drugs used in diabetes**										
	A10BB	Sulfonylureas	287.5	293.5	290.4	13.1	13.3	13.2	41.4	41.2	41.3
	A10BD	Combinations of oral blood glucose lowering drugs	246.1	253.4	249.8	14.4	12.9	13.7	54.4	54.0	54.2
	A10AB	Insulins and analogues for injection. fast-acting	242.1	227.2	234.5	11.0	11.1	11.0	364.5	340.5	352.3
	A10BA	Biguanides	184.9	176.5	180.9	12.1	12.5	12.3	29.4	28.6	29.0
**C08**	**Calcium channel blockers**										
	C08CA	Dihydropyridine derivatives	303.4	273.5	288.2	14.8	17.6	16.2	61.2	59.6	60.4
**N06**	**Psychoanaleptics**										
	N06AB	Selective serotonin reuptake inhibitors	204.6	211.2	209.0	27.9	23.7	25.1	57.3	59.2	58.6
	N06AA	Non-selective monoamine reuptake inhibitors	85.2	74.8	77.9	39.4	39.2	39.2	35.2	31.3	32.5
	N06AX	Other antidepressants	98.4	109.8	105.9	28.8	25.5	26.6	109.9	111.4	110.8
**C03**	**Diuretics**										
	C03DA	Aldosterone antagonists	115.9	112.6	114.1	24.2	25.3	24.8	43.4	40.2	41.7
	C03CA	Sulfonamides, plain	161.3	133.0	144.2	37.1	39.4	38.5	12.7	11.5	11.9
**B03**	**Antianemic preparations**										
	B03BB	Folic acid and derivatives	50.3	61.0	58.0	45.9	53.5	51.4	18.0	15.4	16.1
	B03AA	Iron bivalent, oral preparations	48.9	42.6	44.0	58.8	63.9	62.8	16.8	14.6	15.1
**H03**	**Thyroid therapy**										
	H03BB	Sulfur-containing imidazole derivatives	138.2	137.0	137.3	36.4	37.1	36.9	15.0	14.8	14.9
	H03AA	Thyroid hormones	162.9	147.4	150.0	22.3	18.6	19.3	18.3	17.9	17.9
**N02**	**Analgesics**										
	N02AE	Oripavine derivatives	32.8	27.4	29.0	50.5	49.7	49.9	138.0	128.8	131.5
	N02CC	Selective serotonin (5HT1) agonists	33.5	37.6	36.6	48.7	40.8	42.7	118.2	106.5	109.4
	N02AX	Other opioids	15.2	16.3	15.9	66.1	60.7	62.4	55.5	67.5	63.6
	N02BA	Salicylic acid and derivatives	9.7	10.0	9.8	78.3	78.7	78.6	18.1	18.4	18.3
	N02AB	Phenylpiperidine derivatives	78.0	57.3	64.9	41.5	43.2	42.6	543.3	332.9	410.8
	N02AA	Natural opium alkaloids	18.2	15.5	16.4	59.4	57.0	57.8	111.8	108.0	109.3
**N03**	**Antiepileptics**										
	N03AG	Fatty acid derivatives	126.2	102.5	115.4	15.4	16.8	16.0	146.7	119.6	134.4
	N03AX	Other antiepileptics	97.8	82.8	88.6	32.0	31.7	31.8	195.6	160.7	174.2
	N03AA	Barbiturates and derivatives	273.3	256.3	264.8	12.2	12.9	12.6	25.4	23.4	24.4
	N03AE	Benzodiazepine derivatives	44.3	34.4	39.1	29.7	32.9	31.4	20.8	17.2	18.9
	N03AF	Carboxamide derivatives	201.2	171.7	186.4	13.9	18.0	16.0	113.2	97.0	105.0
